# Evidence That Oscillations in Glucose Metabolism Promote Optimal Islet Function

**DOI:** 10.3390/metabo16040264

**Published:** 2026-04-14

**Authors:** Brian P. List, Nicholas B. Whitticar, Kathryn L. Corbin, Craig S. Nunemaker

**Affiliations:** 1 Department of Biomedical Sciences, Heritage College of Osteopathic Medicine, Ohio University, Athens, OH 45701, USA; bl314720@ohio.edu (B.P.L.); nicholas.whitticar@duke.edu (N.B.W.); corbink1@ohio.edu (K.L.C.); 2 Translational Biomedical Sciences Graduate Program, Ohio University, Athens, OH 45701, USA; 3 Diabetes Institute, Heritage College of Osteopathic Medicine, Ohio University, Athens, OH 45701, USA

**Keywords:** pulsatile, pulsatility, oscillations, oscillatory, D-mannoheptulose, intracellular calcium

## Abstract

**Background/Objectives:** Impairment in pulsatile insulin release contributes to insulin resistance and is one of the earliest markers of developing type 2 diabetes. Insulin delivered to the liver in pulses has a stronger glucose-lowering effect than continuous insulin delivery. Whether pulsatility benefits the islet itself is an open question. We previously showed that reducing glucokinase activity with the glucokinase inhibitor D-mannoheptulose (MH) improves function in islets exposed to prolonged hyperglycemic conditions. In this study, we test whether pulsatile vs. continuous delivery impacts the effectiveness of MH in islets. **Methods:** Islets were exposed to high-glucose conditions (20 mM glucose) for 24 or 48 h to induce early adaptations to hyperglycemia. We then used a specially designed perifusion system to impose pulsatile activity by exposing mouse islets to 3 min of MH in 20 mM glucose and 3 min of only high levels of glucose. Islets given intermittent MH for 18 h were compared with continuous delivery of MH at a full (2.5 mM) or half (1.25 mM) dose. **Results:** MH delivered by the forced oscillatory system reversed the effects of hyperglycemia and restored glucose sensing more effectively than continuous delivery. Specifically, fura-2AM imaging of intracellular calcium showed that islets given pulsatile MH had greater reductions in the elevated basal calcium caused by hyperglycemic conditions, improved the glucose stimulation index, and improved phase 0 response (indicating glucose-stimulated calcium uptake by the endoplasmic reticulum). **Conclusions:** These findings suggest that the loss of oscillatory glucose metabolism in islets contributes directly to beta-cell dysfunction.

## 1. Introduction

Beta cells [1] secrete insulin in a dose-dependent manner proportionally to the levels of glucose in the blood. To accomplish this, the specialized metabolism of glucose in beta cells quantifies the levels of blood glucose and directly stimulates the required level of insulin secretion. This process is termed glucose-stimulated insulin secretion (GSIS). Factors like a chronic high glycemic load (hyperglycemia) and insulin resistance can lead to chronic high levels of insulin secretion (hyperinsulinemia). This leads to an adaptive increase in glucose sensitivity in beta cells, known as a “left shift” in glucose sensitivity, resulting in more insulin with less glucose stimulation [2,3,4,5,6,7,8].

A consequence of this adaptation is the disruption of normal beta-cell physiology, most notably a loss of the pulsatile manner of insulin secretion. An impairment in pulsatile insulin release due to changes in beta cell function is one of the earliest markers of developing type 2 diabetes [9,10,11,12,13]. At the physiological level, insulin-responsive organs like the liver have been shown to respond more effectively to pulsatile insulin, resulting in decreased rates of gluconeogenesis and lipolysis and increased rates of glycogen formation [14,15,16,17]. Paradoxically, the adaptations to increased insulin secretion from beta cells have been shown to decrease glucose uptake in insulin-sensitive tissues, due at least in part to the loss of pulsatile insulin secretion [18].

In beta cells, insulin pulse generation has been modeled as a complex interplay between glycolytic flux, mitochondrial metabolism, and cytosolic free calcium [19]. The pulses generated from glycolytic flux are predominately formed from interactions of the enzyme phosphofructokinase (PFK) [19]. Its product, fructose 6 phosphate (F6P), is a positive feedback regulator resulting in a quick cycling through F6P and buildup of fructose 1,6 bisphosphate (FBP) [19]. AMP increases the affinity of PFK for F6P. Thus, when ATP is produced downstream in mitochondrial metabolism, AMP levels fall. Subsequent turnover of the PFK substrate F6P activity falls, producing a regular oscillation in glycolytic metabolite levels [19]. Mitochondrial metabolism has alternating Mito-Cat and Mito-Ox phases that characterize its influence on pulse generation [20,21,22]. During Mito-Cat, pyruvate kinase (PK) activity is allosterically accelerated through increased FBP levels, which increases the local ATP/ADP ratio, opening K_ATP_ channels and depolarizing the cell until local ADP is depleted. During Mito-Ox, calcium influx causes an increase in ATP-to-ADP turnover that returns the ratio to baseline and closes the K_ATP_ channels [20,21,22]. The calcium-generated pulses result from the negative feedback calcium has on its own entry, signaling its uptake by the endoplasmic reticulum and its stimulatory effects on pyruvate dehydrogenase (PDH) activity, connecting it with both glycolytic efflux and mitochondrial influx [20,21,22]. Together, these mechanisms produce regular insulin pulses, whereas disruptions in glycolysis, mitochondrial function, or calcium handling can impair these oscillatory processes.

Continued chronically elevated GSIS pathway activity eventually leads to beta cell exhaustion and loss of function [8,23,24,25,26]. If significant numbers of functional beta cells are lost, insufficient insulin occurs, and T2D ensues. Importantly, a strong body of evidence indicates that reducing GSIS to normal physiological levels during the initial adapted phase allows the beta cells to rest and can restore normal function [8,25,27,28,29,30,31,32,33,34,35]. This can be accomplished by reducing the glycemic load on the beta cells with caloric restriction and exercise, or through small-molecule inhibitors that target points along the GSIS pathway to reduce activity [8,25,27,28,29,30,31,32,33,34,35]. These studies show the potential for introducing “beta cell rest” as a possible therapeutic intervention.

However, an underexplored aspect of this intervention is the role of pulsatility. Pulsatile insulin has been observed in the blood stream of both mice [36] and humans [37] at ~5 min intervals. In vitro, oscillations were first reported in islets for electrical activity with a period of 4–5 min [38]. Subsequent studies have reported endogenous pulsatile activity in rodent islets with periods ranging from 3 to 6 min for measurements of oxygen consumption [39,40], mitochondrial membrane potential [41,42], and NAD(P)H [43]. Oscillatory activity in islets appears maintain this steady period, with information being coded by amplitude modulation (larger or smaller pulses) as opposed to frequency modulation used in some endocrine systems [13,44,45,46]. Oscillatory activity has also been detected in human islets, primarily using measurements of intracellular calcium [47,48,49,50], with one study even documenting a decline in coordinated activity from human islets with age [48]. However, none of these studies explicitly examined the impact of pulsatility on islet viability or function. The closest such study to date compared mouse islets that could generate endogenous calcium oscillations in 11 mM glucose (11G) to islets that could not; the non-oscillatory group showed significant signs of ER stress and diminished glucose sensitivity [51]. Although in this study, the authors categorized mouse islets as having either pulsatile or non-pulsatile phenotypes and then characterized their respective function. This begs the question: Were the non-pulsatile islets dysfunctional because of their non-pulsatile phenotype or were they non-pulsatile because they were dysfunctional? Since there is little work on this subject, characterizing the effect of non-oscillatory activity on beta cells requires further investigation.

The lack of research into the impact of pulsatility on beta cells is due in large part to the intricacies of pulsatility. Inhibiting any part of the GSIS pathway eliminates pulsatility. Restoring endogenous pulsatility in these conditions requires an intermittent intervention. In this study, islets were exposed to prolonged hyperglycemic conditions (20 mM glucose) to induce early adaptations to hyperglycemia, including impaired pulsatility. We then used a specially designed perifusion system to impose intermittent metabolic activity in beta cells with 3 min on-and-off delivery of the glycolytic inhibitor D-mannoheptulose (MH). Our results showed that the MH delivered by the forced oscillatory system reversed the effects of hyperglycemia and restored glucose sensing more effectively than continuous delivery. Our findings suggest that pulsatile activity is intrinsically important to beta cell health.

## 2. Materials and Methods

### 2.1. Animals

Mice were used as a source of pancreatic islets for these studies. The mice used in the experiments were adult CD1 males ages 10–20 weeks of age, purchased from Envigo (Indianapolis, IN, USA). All experiments involving animals were approved by the Ohio University Institutional Animal Care and Use Committee and were carried out in accordance with the agreed-upon protocol (No. 15-H-021).

### 2.2. Mouse Islet Isolation

For use in the experiments, CD1 mouse islets (pancreatic micro-organs that contain insulin-producing beta cells) were isolated using a protocol consisting of collagenase injection and removal of the pancreas, homogenization, and gradient separation [52]. After the isolation procedure, the islets are placed in a 100 mL petri dish with 11 mL RPMI 1640 medium (Invitrogen, Carlsbad, CA, USA) containing 10% FBS and 1% penicillin/streptomycin and incubated at 37 °C and 5% CO_2_ for two hours to recover. They then undergo two procedures to separate islets from acinar tissue, consisting of pipetting islets to a fresh plate and leaving acinar tissue. The islets are then incubated for a total of 8 h post isolation before treatments are applied.

### 2.3. Islet Pretreatments

Sets of 30 mouse islets are separated from the 100 mL dish into a 12 well plate with 1 mL of standard RPMI 1640 medium along with a treatment condition and incubated for 24–48 h. A total of 11 mM glucose (noted as 11G) media is used to simulate a healthy control, and one pre-treatment group of 11G is required per replicate. A total of 20 mM glucose media (noted as 20G) is used to simulate hyperglycemia and can induce adaptive dysfunction in islets when they are incubated for >24 h; three pretreatment groups in this media are required per replicate.

### 2.4. Oscillatory Drug Platform

**Figure 1:**
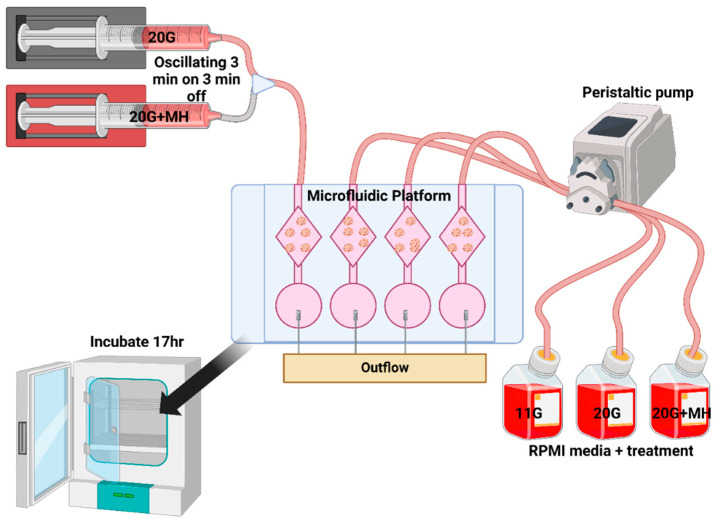
Schematic of the forced pulsatility system: a microfluidic platform, syringe pump, and peristaltic pump used to provide oscillating fluid delivery for studies of islet pulsatility.

Our lab designed a custom microfluidic platform and oscillatory media delivery system to directly look at the effects of endogenous pulsatile activity on beta cell health and function. The system consists of a microfluidic chip, microfluidic tubing, a peristaltic pump, media wells and an oscillatory media delivery system run on computer software, as shown in Figure 1. This pulsatile system is based on a previously published design [53] but with a more sophisticated 3D-printed microfluidic chip. The custom microfluidic chip contains the islets and is connected with tubing on either end to pull media into the main well from a stock of fresh media, or outflow tubing that pulls old media to a waste container. The tubing is connected to a peristaltic pump to drive flow to/from the islet wells and maintain consistent levels. The oscillatory media delivery system consists of two syringe pumps connected to a computer with software that controls the timing interval and speed at which the syringes deliver media. Any treatment condition can be achieved in the circulating media or syringe pump, depending on the type of medium that is used with the microfluidic inflow tubes or syringe pumps. The microfluidic platform can be placed in an incubator for islet treatments to maintain long-term culture conditions as needed.

### 2.5. Forced Pulsatile Activity

**Figure 2:**
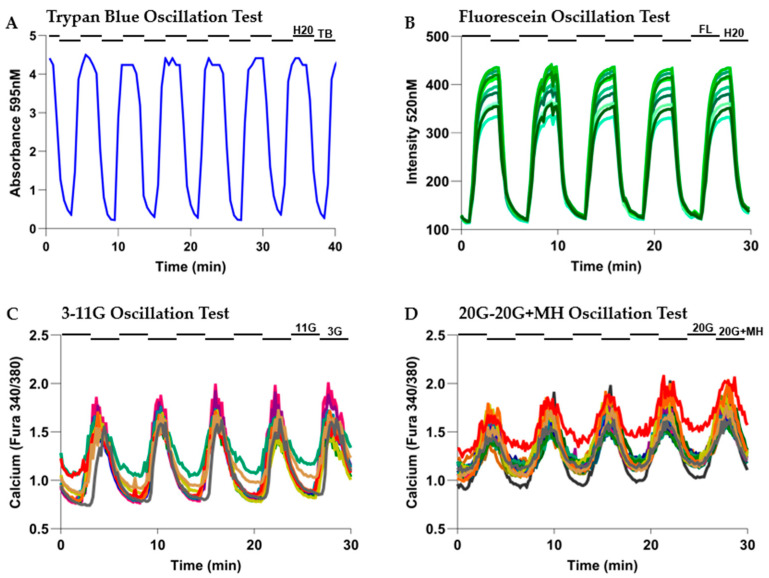
The forced pulsatility system delivers pulses effectively as intended. (**A**) Outflow of the microfluidic plate collected in wells of a 96-well plate every 30 s over 40 min during 3 min media alternations of dH20 or dH20 + 0.08% Trypan Blue with absorbance measured at 595 nm). (**B**) Fluorescence intensity (488 nm/520 nm Em/Ex) taken at 10 different regions in the plate chamber every 10 s during 6 min cycles of dH20 or dH20 + 100 nM fluorescein (3 min for each). (**C**,**D**) Intracellular calcium measured by fura-2AM at 10 s intervals in mouse islets during 6 min cycles of 11 mM or 3 mM glucose media ((**C**), N = 20 islets) or during 6 min cycles 20 mM or 20 mM + 2.5 mM D-Mannoheptulose ((**D**), N = 20 islets). Inflow media changes are indicated with the black bars above the graphs. Lag times for media changes reaching the chamber and time needed for biological effect may affect the timing of oscillations; the 6 min peak-to-peak period indicates correct pulse delivery despite this phase shift.

Previous experimentation in our lab found that 2.5 mM D-Mannoheptulose when used in 20G reduces the level of activity in the islets to that of the 11G control, restoring the islets to a normal state of activity. To induce pulsatile activity, we circulated either 20G + 2.5 mM MH or plain 20G media while on the microfluidic platform. We forced pulsatile activity in the treatment group with 3 min on-and-off delivery of each type of media. During the 3 min of MH, there will be less stimulation to secrete insulin, and during the 3 min of non-MH media, there will be greater drive to secrete insulin. The syringe pumps will cycle with this timing, effectively forcing the beta cells into oscillations in activity level. In humans, insulin pulses measured via the hepatic portal vein have around 5 min periods [37]. The period of insulin pulses has been reported to range from 6 to 8 min when human islets are cultured ex vivo [54]. in our study, 6 min periods were chosen as it is close enough to represent both values. Islets from mice show a similar pulse pattern ex vivo, with various measurements of pulsatility ranging from ~3 to 8 min [42]. We used a period of 6 min (3 min peak stimulation followed by 3 min inhibition) as a physiologically reasonable choice within this range [18]. We did not test other periods as glucose-responsive effects are coded by amplitude rather than frequency (period), which is steady within an individual [13,45,46]. To test this concept, we ran initial experiments (Figure 2D) to see if the oscillatory MH (OSC) delivery could induce calcium oscillations.

### 2.6. Platform-Based Treatment Conditions

After the pretreatment, we placed the 4 groups into the setup (detailed in Figure 1): 11G, 20G, MH and OSC (oscillatory MH delivery). The 11G group started with the >24 h incubation in 11G media followed by a 17 h incubation in circulating (200 μL/min) 11G media while on the microfluidic platform. Thus, 11G serves as a healthy control treatment. The 20G group started with a >24 h incubation in 20G media followed by a 17 h incubation in circulating 20G media while on the microfluidic platform. The 20G group serves as a hyperglycemic control treatment. MH started with a >24 h incubation in 20G media followed by a 17 h incubation in circulating 20G + 2.5 mM (or 1.25 for one trial) MH media while on the microfluidic platform. MH serves as a beta cell rest control as its only difference from the OSC treatment is its lack of oscillatory delivery, whether or not islet activity is in a pulsatile manner. OSC started with a 48 h incubation in 20G media followed by a 17 h incubation in circulating 20G + 2.5 mM MH or plain 20G media while on the microfluidic platform. Differences between the OSC and MH treatments show the effect of pulsatile activity on beta cell health, as theoretically it should be the only difference between the treatment conditions. The 11G and 20G treatments can be used to benchmark these differences.

### 2.7. Glucose-Stimulated Calcium Response

The effects of the treatments were quantified by measuring the glucose-stimulated calcium response (GSCa) using fluorescence microscopy. First, islets were incubated in 2 µM fura-2 AM (Life Technologies, Eugene, OR, USA) for 30 min. Fura-2 AM is a dye that binds to intracellular calcium and is excited with 340 nm light when bound to calcium or 380 nm when it is unbound. After the initial incubation in fura-2 AM, the islets were recorded for fluorescence changes. Islets were imaged using a Hamamatsu ORCA-Flash4.0 digital camera (Hamamatsu Photonics K.K., Hamamatsu City, Japan, Model C11440-22CU) mounted on a BX51WIF fluorescence microscope with a 10× objective (Olympus, Tokyo, Japan). Excitation light was provided by a xenon burner supplied to the image field through a light pipe and filter wheel (Sutter Instrument Co., Novato, CA, USA, Model LB-LS/30) with a Lambda 10-3 Optical Controller (Sutter Instrument Co., Novato, CA, USA, Model LB10-3-1572). Images were taken sequentially with 340 nm and 380 nm excitation for 200 ms exposure every 10 s to produce each intracellular calcium ratio from emitted light at 510 nm. Data were analyzed using cellSens Dimension 1.13 imaging software (Olympus, Tokyo, Japan). Every treatment was paired with a set of 15 islets treated with Cell Tracker Red (CTR) (Invitrogen, Carlsbad, CA, USA), which is an inert fluorescent used to compare each treatment to an 11G untreated control (also treated in fura-2 AM for 30 min). The untreated 11G islets act as a consistent point of reference for each treatment group they are paired with during the recordings [55]. This ensures factors like fluid level, speed, and time for increased glucose to reach the chamber are constant for each treatment group. Increasing glucose increases the fura 340-to-380 nm ratio, which indicates increased calcium intake into the cell. For these studies, islets were recorded initially in KRB solution containing 3 mM glucose (low glucose, noted as 3G) for 4 min, then changed to 8 mM glucose (intermediate glucose, noted as 8G) for 8 min, and finally 20 mM glucose (high glucose, noted as 20G) for the remainder of the experiment (8 min).

### 2.8. Statistical Analysis

**Figure 3:**
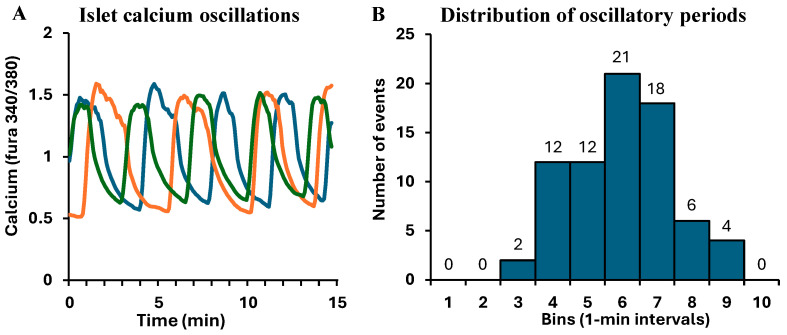
Endogenous calcium oscillations in mouse islets. (**A**) Examples of intracellular calcium patterns recorded in steady-state 11G from three islets that were isolated from one mouse. (**B**) Histogram of the periods of calcium oscillations calculated for N = 75 islets recorded from nine different mice on nine separate occasions. Data were binned at 1 min intervals, with bin 1 consisting of periods from 0–59 s, bin 2 from 60–119 s, bin 3 from 120–179 s, etc. The distribution produces a mean of 5.4 ± 1.4 min, with a skewness of −0.07 and kurtosis of −0.3.

All data were analyzed via a one-way ANOVA with Tukey’s post hoc test unless otherwise indicated, with alpha pre-established as <0.001. *p*-values < 0.001 are indicated as significant (*). Islets differ widely in size, composition, architecture, and function, so islets are generally accepted as individual samples. For example, the distribution of islet values for basal calcium in 3G for the 11G control group ranged from 0.582 to 0.731, with a mean of 0.658 ± 0.0035, N = 54; low skewness of −0.14 (near symmetrical); and excess kurtosis of −1.31 caused by long tails (excess kurtosis dropped to only 0.08 with two outliers removed). A one-way ANOVA was used to check for variability from trial 1 to trial 2 to trial 3 for the 11G control condition; no significant differences were observed for any range of calcium measurements (0–4 min for 3G, 4–12 min for 8G, and 12–20 min for 20G). Statistical comparisons are also reported in the Results by trial (N = 3 per condition) using a one-way ANOVA. The data presented in Figure 3 were derived from a previously published dataset [56] with new representative examples and analysis to show distribution of periods by histogram analysis, which has not been previously reported for this dataset.

## 3. Results

### 3.1. Experimental Setup Testing

A custom perifusion system that includes a dual-syringe pump and a small-volume fluid chamber for perfusing islets was used to conduct these studies (see Figure 1). As shown in Figure 2, several experiments were conducted to test if the system worked as intended. Consistent washout of media with no pooling in the plate and the ability to force calcium oscillations in the islets were verified.

Proper circulation of media (Figure 2A,B) was verified based on consistent amplitudes, which indicates that the dyed solution was washed out quickly between injections from the oscillatory delivery system. The consistent period of the waves at 6 min peak-to-peak indicates that the pump was delivering media with the consistent 3 min on/off timing as intended. Figure 2B shows similar consistent oscillations for fluorescein from the different regions analyzed by the scope, indicating that there are no turbulent areas of pooling media on the plate. The differences in amplitudes seen at each peak are due to the proximity to the center of illumination.

The results in Figure 2C,D show that the microfluidic system successfully forced consistent calcium oscillations. This is highlighted by the increase in the calcium response when the 11 mM/20 mM glucose media was delivered (Figure 2C) and the reduction in response when the 3 mM/20 mM + 2.5 mM MH glucose media was delivered (Figure 2D). Importantly, the amplitude and period of the responses were consistent throughout the experiment. These results also served as a proof-of-concept experiment for our subsequent experiments involving MH.

### 3.2. Endogenous Calcium Oscillations in Islets: Justifying a 6 min Period for Forced Oscillations

Pulsatility is an endogenous and inherent aspect of pancreatic islet function. Three examples of these endogenous calcium patterns are shown in Figure 3A. These examples were all recorded from islets isolated from a single mouse in steady-state 11G, meaning that the islets were kept at the same glucose concentration as their medium to provide a flat rate of energy exposure. Two things to note about these calcium patterns: (1) the overall appearance of these endogenously generated oscillations is quite similar to the forced oscillations shown in Figure 2C by manipulating glucose between 3 and 11 mM. (2) Endogenous oscillations are not synchronized among isolated islets. Although islets presumably must be entrained in vivo to produce pulses of insulin secreted into the portal vein, synchronized calcium oscillations are not typically observed when islets are recorded in vitro. In contrast, the forced pulsatility system does synchronize this activity.

The peak-to-peak period of these oscillations was ~5–6 min, as indicated by the histogram in Figure 3B. This distribution of oscillatory periods was produced from 75 islets isolated from nine different mice on separate occasions. Over 80% of these islets had periods within the 4–7 min bins with a mean period of 5.4 ± 1.4 min. The skewness (−0.07) and kurtosis (−0.3) were quite low, indicating that this is a symmetrical Gaussian distribution. These data justify the choice of 6 min periods for forced oscillations.

### 3.3. GSCa After Overnight Microfluidic Treatments

Studies evaluating pulsatility are lacking in pancreatic beta cell research due to numerous limiting factors. Accordingly, we developed a perifusion system to force pulsatility to alternate between stimulation and inhibition of the GSIS pathway. Islets were incubated in hyperglycemic conditions for 24 or 48 h to saturate glucose stimulation and eliminate endogenous pulsatility. We then tested whether intermittent delivery of MH restores normal islet function better than continuous delivery.

**Figure 4:**
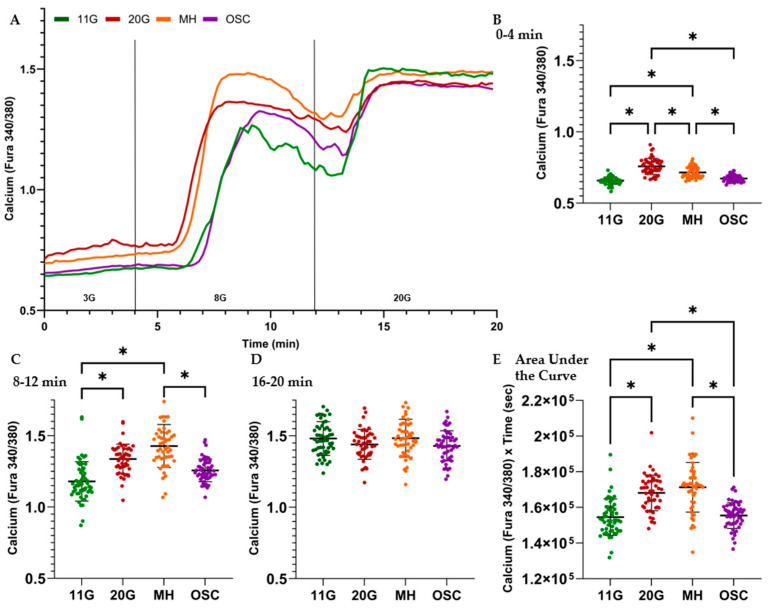
Recordings of intracellular calcium show superior recovery from hyperglycemic conditions in islets given pulsatile MH compared to continuous MH delivery. (**A**) Mean of calcium traces measured by fura-2AM for the islets in each treatment group. Prior to recording, all islets except “11G” were exposed to 20 mM glucose for 48 h pretreatment (24 h in 1 trial). Following the pretreatment phase,”20G” received an additional 24 h of 20 mM glucose treatment; “MH” received a 20 mM glucose + 2.5 or 1.25 mM D-Mannoheptulose (MH) recovery treatment; “OSC” received alternating 20 mM glucose for 3 min and 20 mM + 2.5 mM MH for 3 min recovery treatment. The “11G” islets were maintained in 11 mM glucose throughout as a control. The calcium recording duration was 20 min: 3 mM glucose from 0 to 4 min, 8 mM glucose from 4 to 12 min, and 20 mM glucose from 12 to 20 min. (**B**–**D**). Mean calcium ± SD from 0 to 4 min (**B**), 8 to 12 min (**C**) and 16 to 20 min (**D**) for each islet. (**E**) Total stimulation from 0 to 20 min for each islet is shown, calculated by taking the sum of all recordings × seconds. The recordings were made for three separate trials (multiple islets from one mouse per trial); the individual islet results for a condition were pooled for a total n islets = 54 for 11G, 47 for 20G, 49 for MH, and 53 for OSC. *p*-values were acquired via one-way ANOVA (Tukey’s post hoc), with alpha pre-established as <0.001. *p*-values < 0.001 are indicated as significant (*).

The results in Figure 4A show that calcium levels over time in MH-treated islets tracked more closely with the 20G control than with the 11G control, indicating that the continuous MH treatment did not work as well on its own. Importantly, the opposite was true for the OSC treatment, with calcium levels matching the 11G control more closely than the 20G control. This is statistically supported by the results in Figure 4B,C,E. The basal calcium results in Figure 4B support that the OSC group best recovered the islets from the high-glucose pretreatment, as there was no statistically significant difference between OSC and 11G (*p* = 0.20), with an effect size measured by standardized mean difference (SMD) of 0.61, while there was such a difference between 11G and the 20G control (*p* < 0.001, SMD = 2.38) as well as the MH group (*p* < 0.001, SMD = 1.78). There was also a statistically significant difference between the 20G control and MH (*p* < 0.001), indicating a partial functional recovery though not to the extent of the OSC group, which had significantly improved basal stimulation. These statistically robust observations were supported in trial-to-trial comparisons by a one-way ANOVA (*p* < 0.01 for 11G vs. 20G; *p* < 0.05 for 11G vs. MH; *p* < 0.05 for 20G vs. OSC MH; no other comparisons were statistically significant). These data are in line with islet-level comparisons showing the superior effects of oscillatory MH delivery compared to continuous delivery for restoring basal calcium.

The results in Figure 4C mirror those of the 3G stimulation, with the notable differences being that the MH group had no significant difference (and more surprisingly, higher) stimulation than the 20G control. Again, the OSC treatment restored normal function closest to the 11G control group, with no statistically significant difference. Even in trial-to-trial comparisons, MH differed from 11G (*p* < 0.01) but did not differ significantly from the 20G group. Moreover, continuous MH had significantly higher calcium values in 8G compared to OSC MH, with a strong effect size (SMD = 1.46), providing direct evidence of differences in treatment approaches. Figure 4D shows no statistical differences between any of the groups in islet-to-islet or trial-to-trial comparisons, as they are all maximally stimulated in 20 mM glucose for all conditions.

When examining the area under the curve for calcium throughout the recording, Figure 4E again shows no significant differences between the 11G and OSC groups, while there was a significant improvement (*p* < 0.001) from the OSC group to the 20G and MH groups. Continuous MH treatment, in fact, shows the largest area under the curve, whereas OSC MH treatment shows nearly the same area under the curve as the 11G control condition. Trial-to-trial comparisons showed that the MH group differed from the 11G group (*p* < 0.01) and the OSC MH group (*p* < 0.05); no other significant differences were observed. Collectively, these results indicate that forced pulsatility with MH has a significant beneficial impact on beta cell health that is not observed to the same degree with continuous MH delivery.

### 3.4. Analysis of ER Calcium Handling After Overnight Treatments

**Figure 5:**
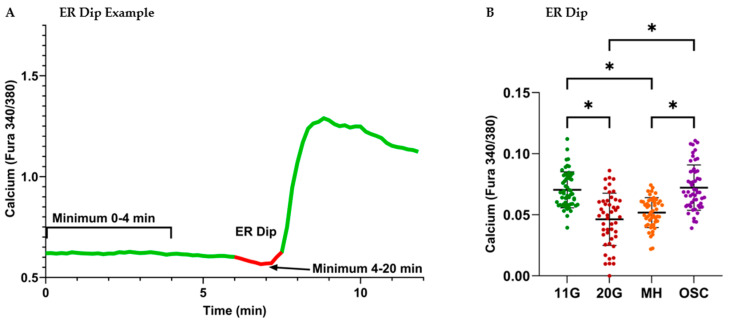
Pulsatile MH delivery enhances the phase 0 (ER dip) islet calcium response to glucose stimulation. (**A**) Representative example of a GSCa recording from a single islet to highlight the phase 0 (ER dip) calcium response. Phase 0 is calculated by subtracting the minimum calcium value after stimulation at 4 min from the minimum calcium value for 0–4 min (basal calcium in 3 mM glucose). (**B**) Calculated phase 0 values for each islet. The recordings (same as in Figure 4) were taken over three trials (none mouse per trial); the individual islet results for a condition were pooled (54 islets for 11G, 47 for 20G, 49 for MH, and 53 for OSC). *p*-values were acquired via a one-way ANOVA (Tukey’s post hoc), with alpha pre-established as <0.001. *p*-values < 0.001 are indicated as significant (*). Data are presented as mean calcium ± SD.

One of the ER’s functions in the beta cell is to store and release intracellular calcium. The ER begins to sequester more calcium when glucose stimulation occurs. In the case of a GSCa assay, the ER starts taking in more calcium when we switch from 3G to 8G, and fura-bound calcium in the ER is not readily detectable. This results in a characteristic ER dip during GSCa recording, where the calcium drops in the cell for a brief moment before KATP channels close to trigger depolarization and subsequent calcium influx through voltage-gated calcium channels. An example of this is shown in Figure 5A.

The results in Figure 5B show the 11G and OSC groups had similarly strong ER dips, whereas 11G and OSC both differed statistically from 20G and MH groups (*p* < 0.001). Both the 20G and continuous MH groups had similarly weak phase 0 (ER dip) calcium responses to glucose stimulation. This suggests that the 20G treatment induced ER stress, and the continuous MH treatment was not able to significantly recover it. However, the OSC treatment was capable of enhancing MH-induced recovery, indicating possible reversal of the hyperglycemia-induced ER stress.

## 4. Discussion

### 4.1. A New Tool for Testing Pulsatility

The pulsatile release of hormones is a feature of many endocrine systems, including the release of insulin into the portal vein leading to the liver. While pulsatility has well-established benefits for hepatic function [14,15,45,57,58], establishing a role for pulsatility in the pancreatic islet has been proven difficult due to numerous limiting factors. Inhibiting one of the key components of stimulus–secretion coupling disrupts not only pulsatility but the secretion pathway itself. The only way to effectively test oscillatory activity in a system is to remove the endogenous pulsatility and restore exogenously imposed pulsatility.

The oscillatory drug platform developed by our laboratory allowed us to directly investigate the role of pulsatile activity on beta cell function using a novel system. In the current study, we demonstrated that the apparatus worked as designed. The wells and pump setup were capable of steady fluid flow and media delivery to the islets. Interestingly, the media clearance rate of our custom wells was greater than a commercially purchased microfluidic chamber. The oscillatory pump was also able to produce consistent and designed media oscillations. As shown in our experiments, having the ability to deliver oscillating media can increase the breadth of possible treatments producing valuable novel findings, and more importantly, it provides a new tool for investigating the roles of pulsatile activity on islet function.

### 4.2. The Importance of Oscillatory Activity to Beta Cell Function

The endogenous oscillatory activity of pancreatic islets is inherently complex, involving biochemical processes of glycolysis and oxidative phosphorylation influenced by large shifts in intracellular calcium driven by ion channels and intracellular stores [59,60]. A large array of endogenous patterns of calcium can be described mathematically by integrated rapid bursting largely driven by electrical activity coupled to slower oscillations in metabolic processes, as described by the Integrated Oscillatory Model [61]. Although our study focused on periodic inhibition of glucokinase with MH, it should be noted that any intervention to similarly reduce other points along the stimulation–secretion pathway should work similarly in principle.

The pulsatile administration of MH showed improved recovery in the induced pulsatile group over the beta cell rest group receiving continuous MH exposure. This suggests that pulsatility is an important factor in beta cell health. From a therapeutic standpoint, pulsatile insulin delivery to the liver has been shown to enhance hepatic function, which is, at least in part, why many automated insulin delivery systems (insulin pumps) deliver insulin in pulses or micro-pulses rather than a continuous stream, often adjusting these micro-doses at 5 min intervals based on continuous glucose monitoring data [62,63]. Though speculative and hypothetical at this point, our data suggest that these 5 min microdoses of MH could have several additional benefits: (1) ER stress in pancreatic islets may be reduced, (2) overall islet health and function may be improved, and (3) more efficient exogenous delivery of insulin may reduce the endogenous drive toward basal hyperinsulinemia by restoring the islet set point (moving the left shift back to the right).

Several recent publications have speculated on the use of small-molecule-induced beta cell rest as a promising avenue for T2D treatment by inhibiting or reducing glucokinase activity [8,25,64,65]. Supporting this idea, glucokinase inactivation improves glucose tolerance in db/db mice by increasing beta cell mass expansion [66]. In a separate study, islets from db/db mice recovered oscillatory function and insulin secretion with a low dose of glucokinase inhibitor [32]. Moreover, the beneficial effects of glucokinase reduction on insulin secretion were linked to restored oscillations in calcium [32]. In a study of human islets preconditioned to hyperglycemia, addition of diazoxide, which opens KATP channels to rest the secretory pathway, could partially restore insulin secretion, pulsatility, and insulin content, again demonstrating the benefits of beta cell rest [27]. As our study suggests, additive improvements to islet function may be possible when the rest is coupled with restoration of oscillatory activity, as shown by the increased recovery of intermittently delivered MH compared to even twice the dose of constant MH. This highlights the potential of pulsatility to increase the effectiveness of therapeutics in treating T2D.

### 4.3. Connecting Pulsatility and the Endoplasmic Reticulum

There have been multiple studies connecting ER stress in beta cells and the development of type 2 diabetes [67,68,69]. The ER is responsible for proper trafficking and processing of proinsulin to insulin. Increases in protein demand lead to an increase in misfolded/unfolded proteins. Unfolded proteins in the ER lumen bind to the molecular chaperone BiP (binding immunoglobulin protein) until they are properly folded [67,68,69]. The preoccupation of BiP causes the disassociation and accompanying activation of three signal-transducing proteins (PERK, ATF6 and IRE1α) from BiP [67,68,69]. The activation of these pathways is known as an unfolded protein response (UPR) [67,68,69]. UPR pathways result in an increase in protein folding capability and degradation of misfolded proteins, as well as a decrease in ER workload and apoptotic factors [67,68,69]. This in turn lowers the stress on the ER and drives it to homeostasis. However, prolonged UPR activation eventually leads to the activation of apoptotic factors leading to programmed cell death [67,68,69].

A physiological marker of ER stress is calcium. When glucose first enters the beta cell and is metabolized, a signal to the ER causes mobilization of calcium from the cytosol. Because calcium taken into the ER cannot be detected with fura-2AM, the drop in cytosolic calcium is reflected by a dip in fura-2AM signal until ATP production reaches a threshold to close KATP channels and thus trigger the large phase 1 spike in calcium entry via voltage-gated calcium channels. When the ER is stressed, it has a decreased ability to sequester calcium; therefore, islets with stressed ER will have reduced or no ER dip. This well-established phenomenon was first noted by its loss in a db/db mouse model of obesity and type 2 diabetes and attributed to ER dysfunction [70,71]. Many studies have confirmed this link between the ER and the initial calcium response to glucose stimulation (phase 0 or ER dip), some of which are included here [68,69,72,73,74], making the phase 0 calcium response a reliable marker of ER function.

Forcing oscillations with MH showed a restoration of a phase 0 response, pointing to a possible reversal of hyperglycemia-induced ER stress. Based on this potential finding and the established understanding of beta cell function, we propose that pulsatile insulin secretion allows time for proinsulin to be properly processed into insulin in the ER. In beta cells, insulin makes up roughly half of all protein processed by the ER [75,76]. Normally, up to 20% of proinsulin fails to achieve proper structure [77,78,79,80]. Increasing glucose can lead to tenfold increases in insulin processing [81] that can increase to fiftyfold during the onset of insulin resistance [82]. The excessive insulin demand from hyperglycemia would require a large amount of protein production from the ER. A lack of pulsatile activity would allow insufficient time for ER processing. This would lead to greater misfolding and cause a buildup of proinsulin in the ER, which could result in ER stress and the activation of the UPR. As insulin production is the dominant protein-folding role of the ER in beta cells, there would be significant accumulation. A constant accumulation of proinsulin would eventually chronically activate the UPR, leading to the activation of apoptotic factors. This would lead to the loss of beta cells, thus contributing to the development of T2D.

We note that more molecular studies are needed to confirm that ER stress is mitigated by periodic bouts of beta-cell rest for ~3 min. Changes in protein or gene expression related to the UPR would be beneficial, but it is difficult to put enough islets into the microfluidics system to check this by conventional qPCR. There are other possible explanations for changes in phase 0 calcium, including certain stressors [72] or shifts in glucose metabolism leading to increased ER calcium in low-glucose conditions, which prevents further changes [70,71,72]. Nevertheless, that small intermittent periods of rest may enhance protein processing is an intriguing hypothesis.

## 5. Conclusions

We have provided proof of concept that oscillations are not only endogenous features of islet function but also a requisite feature of optimal islet health and function. By first saturating oscillatory processes with high glucose and then restoring mid-level activity by reducing glucokinase activity, we showed distinct benefits of stimulating oscillatory activity with intermittent MH compared to continuous MH delivery. If pulsatility indeed contributes to islet health and function, then the loss of pulsatile activity could contribute to beta cell dysfunction during the development of T2D. Additionally, the treatment we used was able to additively restore beta cell function with beta cell rest treatment, increasing the effectiveness of a potential T2D therapeutic. The potential links that we established between pulsatility and the ER will be further examined in future studies to determine if cycles of activity and rest are required for optimal ER function and insulin processing.

## Data Availability

Data are contained within the article or Supplementary Materials.

## Supplementary Materials

The following supporting information can be downloaded at: https://www.mdpi.com/article/10.3390/metabo16040264/s1, ListBP-Metabolites Dataset.

## Abbreviations

The following abbreviations are used in this manuscript:

3G	3 mM glucose
8G	8 mM glucose
11G	11 mM glucose
20G	20 mM glucose
MH	D-Mannoheptulose
OSC	Oscillatory MH delivery
GSCa	Glucose-stimulated calcium response
ER	Endoplasmic reticulum
T2D	Type 2 diabetes
UPR	Unfolded protein response
